# Automated Diagnosis and Grading of Diabetic Retinopathy Using Optical Coherence Tomography

**DOI:** 10.1167/iovs.17-23677

**Published:** 2018-06

**Authors:** Harpal Singh Sandhu, Ahmed Eltanboly, Ahmed Shalaby, Robert S. Keynton, Schlomit Schaal, Ayman El-Baz

**Affiliations:** 1 Department of Ophthalmology and Visual Sciences, University of Louisville, Louisville, Kentucky, United States; 2 Department of Bioengineering, University of Louisville, Louisville, Kentucky, United States; 3 Department of Ophthalmology and Visual Sciences, University of Massachusetts Medical School, Worchester, Massachusetts, United States

**Keywords:** diabetic retinopathy, machine learning, OCT, deep fusion classification networks, neural networks, NPDR, DFCN, SNCAE

## Abstract

**Purpose:**

We determine the feasibility and accuracy of a computer-assisted diagnostic (CAD) system to diagnose and grade nonproliferative diabetic retinopathy (NPDR) from optical coherence tomography (OCT) images.

**Methods:**

A cross-sectional, single-center study was done of type II diabetics who presented for routine screening and/or monitoring exams. Inclusion criteria were age 18 or older, diagnosis of diabetes mellitus type II, and clear media allowing for OCT imaging. Exclusion criteria were inability to image the macula, posterior staphylomas, proliferative diabetic retinopathy, and concurrent retinovascular disease. All patients underwent a full dilated eye exam and spectral-domain OCT of a 6 × 6 mm area of the macula in both eyes. These images then were analyzed by a novel CAD system that segments the retina into 12 layers; quantifies the reflectivity, curvature, and thickness of each layer; and ultimately uses this information to train a neural network that classifies images as either normal or having NPDR, and then further grades the level of retinopathy. A first dataset was tested by “leave-one-subject-out” (LOSO) methods and by 2- and 4-fold cross-validation. The system then was tested on a second, independent dataset.

**Results:**

Using LOSO experiments on a dataset of images from 80 patients, the proposed CAD system distinguished normal from NPDR subjects with 93.8% accuracy (sensitivity = 92.5%, specificity = 95%) and achieved 97.4% correct classification between subclinical and mild/moderate DR. When tested on an independent dataset of 40 patients, the proposed system distinguished between normal and NPDR subjects with 92.5% accuracy and between subclinical and mild/moderate NPDR with 95% accuracy.

**Conclusions:**

A CAD system for automated diagnosis of NPDR based on macular OCT images from type II diabetics is feasible, reliable, and accurate.

Diabetes is a chronic, systemic disease with an estimated prevalence of 29 million in the United States and over 400 million worldwide.^1,2^ Its microvascular complications are well known, including diabetic retinopathy (DR), from which an estimated 38% of diabetics suffer worldwide.^3^ Early detection and treatment of the disease are mainstays of management, and this principle drives the American Academy of Ophthalmology and American Diabetes Society guidelines for annual dilated fundus exams for all type 2 diabetics at diagnosis and type 1 diabetics 5 years after diagnosis.^4,5^ The growing epidemic of diabetes in the United States poses the particular public health challenge of screening an ever growing number of diabetics. Telescreening has been valuable in this regard, but suffers from variable image quality and subjective interpretations.^6^

Traditionally, diagnosis of DR has been clinical with adjunctive testing, such as fluorescein angiography (FA) and optical coherence tomography (OCT), used to confirm or quantify clinical suspicion of structural complications, such as neovascularization and macular edema. Except for common metrics, such as central macular thickness or macular volume, the interpretation of OCTs in diabetics has been predominantly subjective. To the best of our knowledge, an accurate, automated screening system for DR based on OCT does not exist. Such a system would fulfill the dual goals of reducing the burden of diabetic screening for clinicians and reducing the subjectivity of OCT interpretation. For nonproliferative diabetic retinopathy (NPDR) screening, we aimed to create a computer-assisted diagnostic (CAD) system to automatically differentiate between normal retinas and those with mild or moderate NPDR. A second goal was to determine if there were subclinical OCT changes in diabetic patients without overt NPDR on exam, a condition we called subclinical DR. With the recent improvement in artificial intelligence and its myriad applications to medical imaging, we sought to apply machine learning techniques to this endeavor. Machine learning is a branch of computer science that enables machines to learn to perform a task, such as diagnosing and grading OCTs for DR, without having been explicitly programmed for such an end. These techniques are well suited to image analysis, as they potentially can make diagnoses based solely on complex images, such as computed tomography (CT) and magnetic resonance imaging (MRI) scans or OCTs. While the initial medical applications naturally started in radiology, ophthalmologists recently have begun to apply these powerful tools to our field, which is replete with imaging. Fundus photographs, of which there are several large, public collections of data, were the first to be subject to machine learning techniques on a large scale. Several diagnostic systems have shown excellent accuracy in classifying images with DR, age-related macular degeneration (AMD), and glaucoma.^7–11^ More recently, these techniques have been extended to OCTs, a nearly ubiquitous technology in retina practices, and this study continues in this vein.

## Methods

This was a cross-sectional study of type II diabetics who presented to the University of Louisville for routine screening and/or monitoring exams in 2014, 2015, and 2018. Inclusion criteria were age 18 or older, diagnosis of diabetes mellitus type II, and clear media allowing for OCT imaging. Exclusion criteria were inability to image the macula, posterior staphylomas (because of confounding effects on OCT images), proliferative diabetic retinopathy (PDR), and concurrent retinovascular disease that might confound diagnosis. A full dilated eye exam was conducted by an attending retina specialist. All patients were diagnosed clinically as having no DR or DR based on dilated fundus exam, with DR further subdivided into the typical clinical categories of mild, moderate, or severe. OCT scans (Zeiss Cirrus HD-OCT 5000; Carl Zeiss Meditec, Inc., Dublin, CA, USA) of a 6 × 6 mm area of the macula of both eyes were performed. Fluorescein angiography was performed only in an ad hoc fashion per the discretion of the attending physician and was not a standard part of the study protocol. This study was approved by the institutional review board of the University of Louisville, and was conducted in accordance with the tenets of the Declaration of Helsinki.

### The CAD System

**Figure 1:**
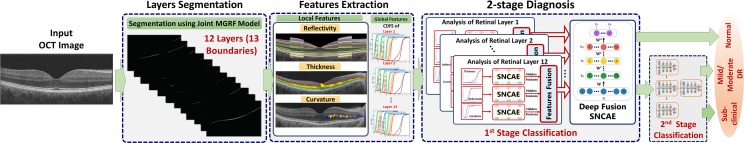
The framework of the proposed approach illustrating the main steps of the system.

A novel noninvasive framework for early diagnosis of DR using OCT images then was developed (Fig. 1) in which three steps are performed sequentially. First, 12 distinct retinal layers are segmented using previously described methods.^12^ In this method, a joint model that integrates morphologic, spatial, and intensity information is adopted. Second, three global features are measured based on curvature, reflectivity, and thickness of the segmented retinal layers. Finally, a two-stage, deep network is used to classify the test subject as normal, or having subclinical or mild/moderate DR. Subclinical DR was defined as when the clinical fundus exam was negative for DR but the OCT demonstrated features intermediate between those of normal and DR cases. The details of the CAD system are summarized.

In brief, the segmentation algorithm is based on a joint model that integrates shape, intensity, and spatial information. First, a set of 12 normal OCTs (from six males and six females) was used as the template for automated segmentation. Second, OCTs from 200 normal patients, aged 18 to 75, were segmented by four different retinal specialists and used as a gold standard, or “ground truth,” for normal OCT images (Supplementary Fig. S1). This was the shape model. Third, the automated segmentation algorithm was applied to the 200 manually segmented normals to ensure accuracy. The intensity model was built using a linear combination of discrete Gaussians (LCDG) model. To account for noise and inhomogeneities, the spatial information is modeled using a second-order Markov Gibbs random field (MGRF).^13^ This approach generates region maps that are close to the gold standard validated by retinal specialists.

**Figure 2:**
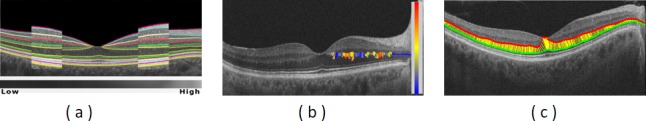
Reflectivity (a), curvature (b), and thickness (c) features are illustrated. (a) The red and blue rectangles illustrate the reflectivity feature obtained from two regions per scan, comprising the thickest portions of the retina on the nasal and temporal sides of the foveal peak. (b) The color map demonstrates how curvature values range from low, colored blue, meaning closest to a straight line, to high, colored red. (c) The yellow bars depict thickness as the distance between two corresponding points at upper and lower boundaries of a given retinal layer.

Three distinct retinal features quantifying reflectivity, curvature, and thickness were extracted from each segmented OCT image (Fig. 1). Reflectivity was obtained from two regions per scan, comprising the thickest portions of the retina on the nasal and temporal sides of the foveal peak (Fig. 2a). The vitreous, which was clear in all patients, was defined as having a reflectivity of 0 and the hyperreflective RPE layer was defined arbitrarily as having a reflectivity of 1000. All other points had a reflectivity between that of the clear vitreous and the hyperreflective RPE and, thus, could be quantified on this 1000-point scale. After smoothing of the image, the curvature of each retinal layer (Fig. 2b) was calculated for each point across the layer. Thickness of the retinal layers was determined by the distances between corresponding points on the lower and upper boundaries of each layer, as is customary (Fig. 2c).

For each subject, these three features were described as a whole with a cumulative distribution function (CDF) of the extracted retinal layers. The CDFs were considered global discriminatory characteristics, able to distinguish between normal and DR cases. In our system, the CDFs for a training set of the OCT images were used for deep learning of a multistage classifier with autoencoders, which are a form of unsupervised learning.

In the final step, after segmenting the 12 retinal layers and extracting the three key features, the CAD system classified normal and DR subjects. Since this data set was large, we used a form of a neural network called a deep learning network that had the ability to learn these features and fuse them together. To learn characteristics of normal and DR subjects, CDFs were calculated for each feature and fed into the proposed network. To build the classification model, a deep neural network with two stages of autoencoders was used.^14^ The first stage consisted of several deep networks built with the encoders for each input feature, one autoencoder for each of the three features per each segmented layer for a total of 36 (12 × 3 = 36). In the second stage, detailed classification of subjects with DR was performed to determine the grade of DR using the deep fusion classification network.

### Statistical Analysis

The sensitivity, specificity, accuracy, and area under the receiver operating curve (AUC) were calculated for the CAD system. Accuracy was defined as true-positives plus true-negatives divided by the total number of cases. The attending physician's final diagnosis by clinical funduscopic exam was considered the gold standard diagnosis and used to calculate the efficacy of the proposed system. The system was tested using a leave-one-subject-out (LOSO) method. This involves training the system on images from *n*-1 eyes and then testing it on the images of the sole eye left out, hence LOSO. This process then is repeated *n* times. It also was tested by 2- and 4-fold cross-validation. In 2-fold cross-validation, each fold contained 40 subjects (20 normal, and 10 subclinical and 10 mild/moderate DR subjects). First, one fold was used for training and one for validation. This operation was repeated several times by changing the validation fold each time to evaluate the accuracy. In 4-fold cross-validation, each fold contained 20 subjects (10 normal, and five subclinical and five mild/moderate DR subjects). First, three folds were used for training and one fold for validation. This operation also was repeated several times by changing the validation fold each time to evaluate the accuracy. Then, 95% confidence intervals (CI) were calculated using the bootstrapping technique.^15^ To evaluate its accuracy, the proposed CAD system was compared to three established systems, or classifiers, based on machine learning. These are state-of-the-art classifiers available in the public domain that can serve as a benchmark for other novel classifiers, such as the system described herein. The three systems used for comparison were from the Weka collection^16^ from the University of Waikato (New Zealand); K*, k-nearest neighbor (kNN); and Random forest (RF). A Dice (Sørensen-Dice) similarity coefficient, a measure of the similarity of two sample sets, was used to compare the system's segmentation performance to expert segmentation.

## Results

To test and validate the proposed segmentation method, OCT scans (Zeiss Cirrus HD-OCT 5000; Carl Zeiss Meditec, Inc.) were collected prospectively from 160 eyes of 80 subjects (52 female, 26 male) 23 to 81 years old (mean, 60; interquartile ratio [IQR], 53–68). All patients had type 2 diabetes mellitus, ranging from <1 year to >30 years since diagnosis, and 33 (41.2%) had systemic hypertension on oral hypotensive agents. Most recent hemoglobin A1c measurements and glomerular filtration rates were not available. Of the 160 eyes, 120 clinically had no DR and 40 had mild or moderate NPDR. Of the 120 without clinical DR, 40 (33%) had OCT changes intermediate between normal retinas and those with mild NPDR. We called this the “subclinical DR” group.

**Figure 3:**
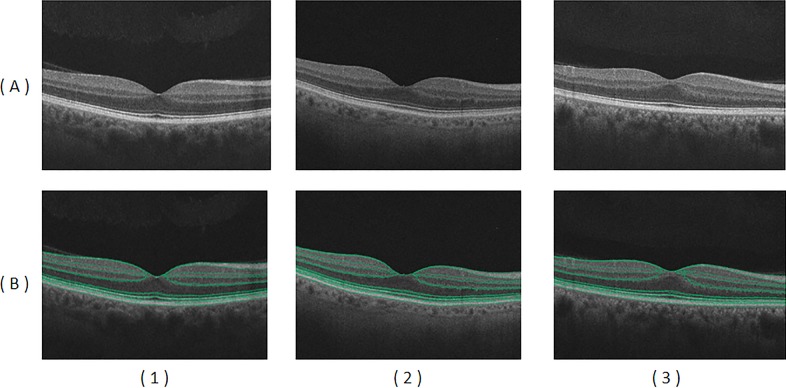
Examples of OCT images in row (A) for normal (1), subclinical DR (2), and mild-moderate DR (3) cases. Results of automated segmentation are displayed in row (B) showing the detected 13 boundaries.

The proposed automated segmentation approach was validated using a gold standard for normal subjects, which was created by manual delineations of retinal layers with the aid of retina specialists. Figure 3 shows different examples for the segmentation of 12 distinct retinal layers. The Dice similarity coefficient metric comparing segmentation results with the gold standard had a mean value of 0.84, and mean boundary error was 6.87 μm from the ground truth, averaged across all 13 boundaries.

Following retinal layer segmentation, our system assessed the grade of DR. A LOSO approach was applied to distinguish first between normal and DR subjects, and then second between subjects with subclinical and mild/moderate DR. The Table presents the relative diagnostic accuracies of the three publicly available classifiers and our proposed system in terms of the number of correctly classified cases with respect to the overall numbers of subjects. For the first stage (normal vs. DR), the proposed CAD system showed a total diagnostic accuracy of 93.8% (150/160 subjects), or, among the 80 total eyes, 74 were classified correctly as completely normal and 76 were classified correctly as abnormal with either subclinical (40 eyes) or mild/moderate (40 eyes) DR. For the second stage, which distinguished subclinical versus mild/moderate DR, total diagnostic accuracy was 97.4%. The receiver operating characteristics (ROC) analysis for the proposed system as well as the three other systems also was calculated. The calculated area under the ROC curve was highest for the proposed CAD system and approached the maximum possible value of 1 (Table). By 4-fold cross-validation, the average accuracy across all four folds was 92.5% for distinguishing normal from DR. For distinguishing subclinical from mild/moderate DR, the average diagnostic accuracy was 94.6%. The 95% CI ranged from 83% to 96%. By 2-fold cross-validation, the average accuracy across all four folds was 90%. For distinguishing between subclinical and mild/moderate DR, the average diagnostic accuracy was 94.5%.

**Table:**
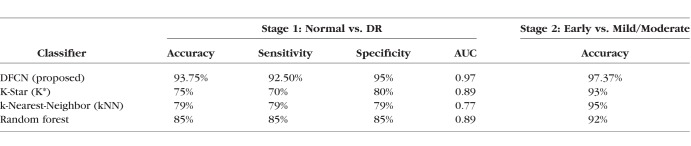
Classification Accuracy, Sensitivity, Specificity, and the AUC for the CAD System Using the Initial Dataset of 80 Patients and for Three Other Common Classifiers From the University of Waikato

Finally, the system was tested on an independent set of OCT images from 80 eyes of 40 patients (55% female; mean age, 54; age range, 21–74; duration of diabetes, 1–21 years). Of these patients, 60 had no DR on clinical exam and 20 had mild or moderate NPDR. The system was trained on all 80 patients from the original dataset before being tested on the de novo set of 40 patients. The proposed CAD system showed a total diagnostic accuracy of 92.5% (74/80 eyes), or 36 of 40 normal eyes classified correctly as completely normal and 38 of 40 with mild/moderate NPDR (20 eyes) or subclinical DR (20 eyes) classified correctly as abnormal. For the second stage (subclinical vs. mild/moderate DR), the proposed CAD system achieved a 95% total diagnostic accuracy.

## Discussion

OCT is a powerful modality for the noninvasive diagnosis of several conditions, including glaucoma, macular edema, choroidal neovascularization, and AMD, another common retinal disease. With diabetic retinopathy, early diagnosis and management are critical to prevent vision loss, but regular screening of the ever-increasing diabetic population in the United States and worldwide is a daunting public health challenge. Automated early detection of diabetic retinopathy can assist in this endeavor, but to date has not used OCT images for diagnosing and grading NPDR. Our system was 94% accurate in distinguishing between the presence or absence of DR, and 97% accurate in distinguishing subclinical DR changes from mild or moderate DR. These results are an encouraging proof-of-concept for automated diagnostics and suggested that the system is reliable and accurate.

Early work in this area has focused on detecting conventional clinical lesions associated with DR.^17–20^ Few have investigated using nonclinical features for DR detection. Recently, an advanced machine learning technique, called deep learning, has been used to classify fundus photographs of DR. This technique involves a highly sophisticated form of artificial intelligence, typically using neural networks, that can learn to perform tasks with minimal input from human programmers and little labeling of the data involved. The power of this approach lies in its potential accuracy and ability to make connections not readily apparent to human investigators. For instance, a recent multicenter study was able to predict cardiovascular risk factors reliably, including age, smoking status, blood pressure, and history of cardiac events, simply from fundus photographs.^21^ The tradeoff is that it requires enormous amounts of data, tens or hundreds of thousands of images, to fully exploit this approach. The aforementioned work for detecting DR has been performed using fundus images. One of its fundamental drawbacks is that it provides images in only two dimensions with no appreciation for depth, compared to OCT imaging which provides quantifiable depth information; therefore, it is possible to detect pathology with topological changes in vivo. To the best of our knowledge, this is the first automated, early detection grading system of NPDR using OCT images. Because there are no analogous, public datasets of OCT images, applying a similar type of deep learning to OCTs in DR is more challenging, but attempts have been made for other retinal diseases.

Machine learning also has been applied recently to OCTs of AMD patients with good success. Normal and age-related macular degeneration images from the Heidelberg Spectralis database (Heidelberg Engineering, Heidelberg, Germany) were analyzed using machine learning, producing AUCs of 94% and 89%,^22^ comparable to our results for NPDR. However, the aforementioned study distinguished only between the presence and absence of AMD and did not grade the disease. A similar study using the European Genetic Database successfully graded AMD with similar accuracy to human graders.^23^

Of the 160 eyes in our first dataset, 40 (25%) had no clinical DR but showed OCT changes in curvature, thickness, and/or reflectivity that were intermediate between normal OCTs and those seen in mild and moderate NPDR. This was called the “subclinical DR” group. Identifying such a group comes as little surprise. It long has been established that diabetics without clinical DR have electrophysiologic abnormalities. Furthermore, they also show changes in thickness of different retinal layers, including thinning of the retinal nerve fiber and ganglion cell layers, and thickening of the inner nuclear and outer plexiform layers.^24–27^ This is consistent with the hypothesis that diabetic retinopathy is not purely a microvascular disease, but also a retina-wide neuropathy.^28^

The principal limitation of our study is its limited number of subjects, a common occurrence in single-center studies. However, despite fewer than 200 patients, this system achieved high levels of accuracy comparable to other CAD systems using OCT to classify AMD and superior to some CAD systems using fundus photographs to classify NPDR. The system's novel segmentation steps are integral to the system as a whole, and our novel classifier also improves upon publicly available ones, albeit modestly. A second limitation is the lack of higher grades of DR, as there were no cases of severe NPDR. In the future, we aim to broaden this approach to include PDR as well.

Recently, further light has been shed on the microvascular changes in DR using OCT angiography (OCTA). Multiple new insights have been gleaned from this emerging technology. Several groups have established that capillary density in the superficial capillary plexus and the deep capillary plexus correlate inversely with the severity of DR.^29,30^ Others have shown that visual acuity in diabetics correlates inversely with the size of the foveal avascular zone.^31,32^ Further research is needed to combine OCT information with that of OCTA to increase the overall accuracy and robustness of future CAD systems.

A final issue to address is the extent to which DR, a retina-wide pathology, can be diagnosed solely with macular imaging. Two points warrant mention. First, to some extent, the results of this study lend credence to the claim that accurate diagnosis can be made solely by OCT of the macula. Second, a litany of studies using fundus photographs of just the posterior pole taken by nonmydriatic fundus cameras have shown good sensitivity, which represents further evidence to this effect.^33,34^ While peripheral DR lesions may carry a slightly greater risk of DR progression over time,^35^ the vast majority of them are posterior. The traditional Early Treatment of Diabetic Retinopathy (ETDRS) seven-fields photography, long the standard for documenting DR in clinical trials, captures only the posterior 30% of the retinal area. Ultrawide field imaging captures a much larger area and much more of the retinal periphery, yet in one large study it detected the presence of DR in only a further 3% of cases over standard ETDRS photography, which speaks to the predominantly posterior location of the disease.^36^

In summary, we proposed a novel CAD system for detection and classification of DR using OCT images. The framework includes a robust approach for segmentation of 12 distinct retinal layers. A two-stage, deep fusion classification network is used to classify subjects as normal, subclinical stage DR, or mild/moderate DR based on three discriminant features, namely curvature, reflectivity, and thickness, across all the segmented retinal layers. This system achieved an average of 94% total diagnostic accuracy. In the future, it could be combined with OCT angiographic data to improve accuracy and ultimately generalize to PDR as well.

## Supplementary Material

Supplement 1
