# Supplementary material for: Automated Diagnosis and Grading of Diabetic Retinopathy Using Optical Coherence Tomography
Source: Invest Ophthalmol Vis Sci. 2018 Jun;59(7):3155–60. doi: 10.1167/iovs.17-23677 (PMC6018370; doi:10.1167/iovs.17-23677)
Supplement: Supplement 1 [file iovs-59-07-08_s01.pdf]

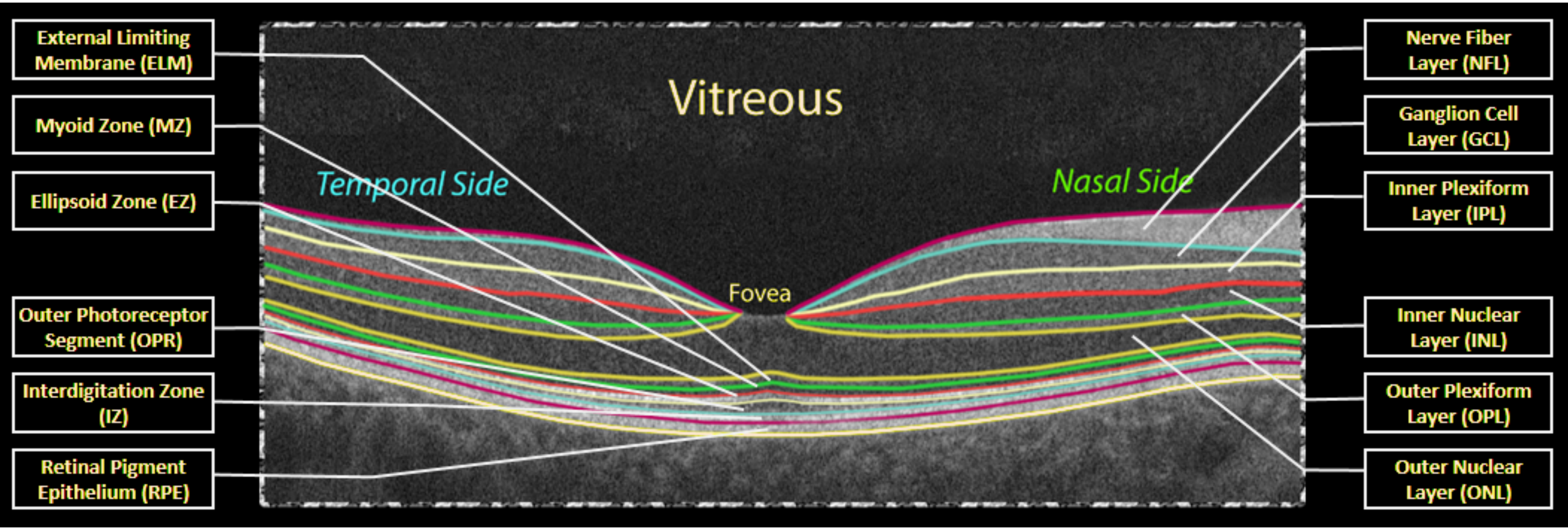

External Limiting Membrane (ELM)
Myoid Zone (MZ)
Ellipsoid Zone (EZ)
Outer Photoreceptor Segment (OPR)
Interdigitation Zone (IZ)
Retinal Pigment Epithelium (RPE)
Vitreous
Temporal Side
Nasal Side
Fovea
Nerve Fiber Layer (NFL)
Ganglion Cell Layer (GCL)
Inner Plexiform Layer (IPL)
Inner Nuclear Layer (INL)
Outer Plexiform Layer (OPL)
Outer Nuclear Layer (ONL)
